# Using controlled disorder to probe the interplay between charge order and superconductivity in NbSe_2_

**DOI:** 10.1038/s41467-018-05153-0

**Published:** 2018-07-18

**Authors:** Kyuil Cho, M. Kończykowski, S. Teknowijoyo, M. A. Tanatar, J. Guss, P. B. Gartin, J. M. Wilde, A. Kreyssig, R. J. McQueeney, A. I. Goldman, V. Mishra, P. J. Hirschfeld, R. Prozorov

**Affiliations:** 1 Ames Laboratory, Ames, IA 50011 USA; 2 Department of Physics and Astronomy, Iowa State University, Ames, IA 50011 USA; 3 Laboratoire des Solides Irradiés, École Polytechnique, CNRS, CEA, Université Paris-Saclay, F-91128 Palaiseau, France; 4 Computer Science and Mathematics Division, Oak Ridge National Laboratory, Oak Ridge, TN 37831 USA; 5 Department of Physics, University of Florida, Gainesville, FL 32611 USA

## Abstract

The interplay between superconductivity and charge-density wave (CDW) in 2*H*-NbSe_2_ is not fully understood despite decades of study. Artificially introduced disorder can tip the delicate balance between two competing long-range orders, and reveal the underlying interactions that give rise to them. Here we introduce disorder by electron irradiation and measure in-plane resistivity, Hall resistivity, X-ray scattering, and London penetration depth. With increasing disorder, the superconducting transition temperature, *T*_c_, varies non-monotonically, whereas the CDW transition temperature, *T*_CDW_, monotonically decreases and becomes unresolvable above a critical irradiation dose where *T*_c_ drops sharply. Our results imply that the CDW order initially competes with superconductivity, but eventually assists it. We argue that at the transition where the long-range CDW order disappears, the cooperation with superconductivity is dramatically suppressed. X-ray scattering and Hall resistivity measurements reveal that the short-range CDW survives above the transition. Superconductivity persists to much higher dose levels, consistent with fully gapped superconductivity and moderate interband pairing.

## Abstract (web summary)

The interplay between superconductivity and charge density wave (CDW) in 2*H*-NbSe_2_ is still not fully understood. Here, Cho et al. use controlled disorder to probe the interplay between these two phases in 2*H*-NbSe_2_ and find that superconductivity initially competes with CDW but eventually long-range CDW order assists superconductivity.

## Introduction

The interplay between superconductivity (SC) and density wave orders has been a central issue in high-temperature superconductors such as cuprates and iron-based superconductors^1^. The recent discovery of a charge-density wave (CDW) phase in the middle of the pseudogap region of cuprates^2–7^ has revitalized interest in the interplay between SC and CDW orders in other unconventional superconductors, such as the layered transition-metal dichalcogenides, in particular the well-studied 2*H*-NbSe_2_^8–11^. This system has fascinated investigators for decades due to the microscopic coexistence of CDW (*T*_CDW_ = 33 K) and SC (*T*_c_ = 7.2 K) states^12,13^. The coupling between the two long-range orders is apparently responsible for the observability of the elusive Higgs bosonic amplitude mode of the superconductor^14,15^, revealed by Raman scattering on 2*H*-NbSe_2_^16,17^.

Despite intense effort, however, a key question regarding the nature of the coupling of the two orders in this system is still under debate. In recent years, the conventional weak coupling picture where CDW and SC compete for parts of the Fermi surface has been challenged by the realization that the electron-phonon coupling is very strong due to the two-dimensional confinement of the Nb layer^10,18–21^. In such a situation, the usual mean field picture of a CDW order with rigid amplitude and phase disappearing at *T*_CDW_ may no longer be valid, as the short-range CDW order together with a gap in the electronic spectrum has been observed outside the long-range ordered phase^22^.

The situation in 2*H*-NbSe_2_ is complicated by the complex electronic bandstructure of this material and apparent multiband SC with two effective gaps^23^. Different superconducting gaps on different Fermi surface sheets were inferred from angle-resolved photoemission spectroscopy (ARPES) by Yokoya et al.^24^ and thermal conductivity measurements by Boaknin et al.^25^. Kiss et al.^26^ proposed that the CDW actually boosts the SC, based on the correlation with the largest electron-phonon coupling and lowest Fermi velocities at the same *k*-points. Borisenko et al.^27^ observed Fermi arcs, suggesting that the CDW inhibits the formation of superconducting order by gapping the nested portion of the Fermi surface.

The pressure dependence of *T*_CDW_ and *T*_c_ is another way to study the interplay between SC and CDW orders. Leroux et al.^28^ suggest that the pressure and temperature dependence of the phonon dispersion, observed by inelastic X-ray scattering, supports the insensitivity of *T*_c_ to the CDW transition. However, Feng et al.^29^ reported a broad regime of order parameter fluctuations in X-ray diffraction (at *T* = 3.5 K) and attributed it to the presence of a CDW quantum critical point (*P*_CDW_ = 4.6 GPa) buried beneath the superconducting dome^29^. They also claimed that this is partially consistent with the increasing *T*_c_ under pressure up to about 4.6 GPa^30^. Suderow et al.^31^ proposed a peculiar interplay among SC, CDW order, and Fermi surface complexity, based on the mismatch between the suppression of *T*_CDW_ at 5 GPa and the maximum *T*_c_ at 10.5 GPa. Chatterjee et al.^22^ studied the effect of transition-metal doping on the CDW state using ARPES, X-ray diffraction, scanning tunneling microscopy (STM), and resistivity, and showed that short-range CDW order with an energy gap survives at high temperatures and high disorder beyond the phase coherence transition.

Another way to probe CDW and SC states is to introduce non-magnetic point-like disorder^32–36^. Electron irradiation, which has been shown to create pure atomic disorder without doping the system as deduced from Hall effect measurements, is an effective tool to influence both the SC and other orders^37,38.^ Moreover, independent simultaneous measurements of *T*_c_, *T*_CDW_, and low-temperature London penetration depth in the same samples with controlled point-like disorder become powerful techniques that can distinguish different types of superconducting pairing such as *d*-wave, *s*_±_, and *s*_++_ pairings^34,35,39^. Indeed, this approach was successfully used in various iron-based superconductors^36,37,40,41^. According to early studies of the effect of electron irradiation on NbSe_2_ by Mutka et al.^32^, an increase of *T*_c_ was reported but attributed to inhomogeneous SC. This result was theoretically discussed by Grest et al.^33^ and Psaltakis et al.^42^, but direct evidence determining the effect of homogeneously distributed disorder on the interplay between CDW and SC states is still missing.

In this study, we systematically investigate the effect of controlled point-like disorder on SC and CDW order in 2*H*-NbSe_2_. The disorder is generated by applying 2.5 MeV electron irradiation with different doses. For each dose, the changes in *T*_c_, residual resistivity, Hall coefficient, and London penetration depth are measured. For low irradiation doses, *T*_c_ shows non-monotonic behaviour, first increasing slightly and then decreasing until a critical dose of 1.0 C cm^−2^ where it drops abruptly. At this critical dose, the long-range CDW feature in resistivity disappears as well. The vanishing of *T*_CDW_ appears to be discontinuous. Upon further irradiation, we find the existence of persistent short-range CDW correlations based on X-ray scattering and Hall resistivity measurements, and attribute the abrupt drop in *T*_c_ to the loss of coherence of the phase-coherent CDW order. Among various possible mechanisms, we conclude that the effect of the reconstruction of the electronic structure by the CDW leads to a rapid change of electron-phonon scattering at the critical dose of 1.0 C cm^−2^, explaining the remarkable qualitative change in the Hall effect and the abrupt drop of *T*_c_. This represents clear evidence for a special role of the coherent CDW state coupling to SC. Furthermore, the change in *T*_c_ provides important information on the nature of the pairing both within and outside of the long-range CDW state. Upon irradiation above the critical dose, *T*_c_ continuously decreases down to the largest dose applied, suggesting a substantial degree of gap anisotropy. The low-temperature London penetration depths of three post-irradiated samples consistently show exponentially saturating behaviour below 0.2 *T*_c_, with gaps that increase with disorder and are therefore consistent with this picture.

## Results

### Effect of electron irradiation on resistivity

**Fig. 1:**
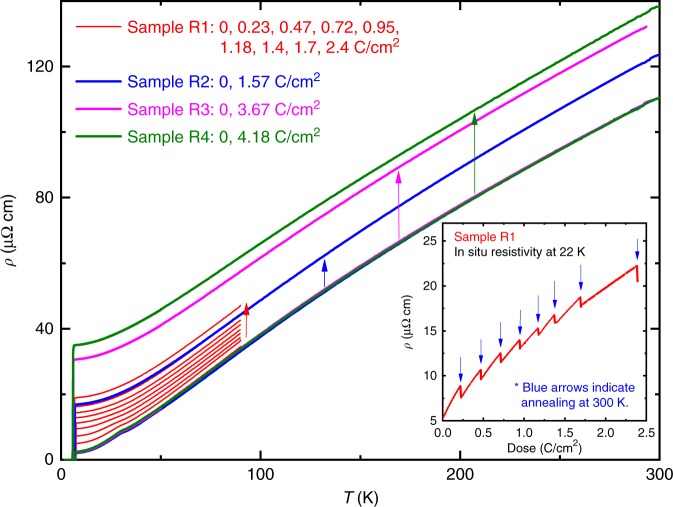
Temperature dependence of resistivity upon electron irradiation. Resistivities of four different samples: R1 (0, 0.23, 0.47, 0.72, 0.95, 1.18, 1.4, 1.7, and 2.4 C cm^−2^), R2 (0, 1.57 C cm^−2^), R3 (0, 3.67 C cm^−2^), and R4 (0, 4.18 C cm^−2^). Note that all 0-dose curves for samples R1, R2, R3, and R4 are coincident. Overall resistivity increase with increasing dose was consistently seen for all samples, as shown by the arrows. The inset shows in-situ measurement of resistivity of sample R1 as a function of dose during electron irradiation at 22 K. The blue arrows indicate stops in irradiation, during which the sample was extracted from the irradiation chamber and characterized. Partial annealing of about 30–40% of resistivity increase occurred on warming the sample to room temperature and subsequently cooling down to 22 K

**Fig. 2:**
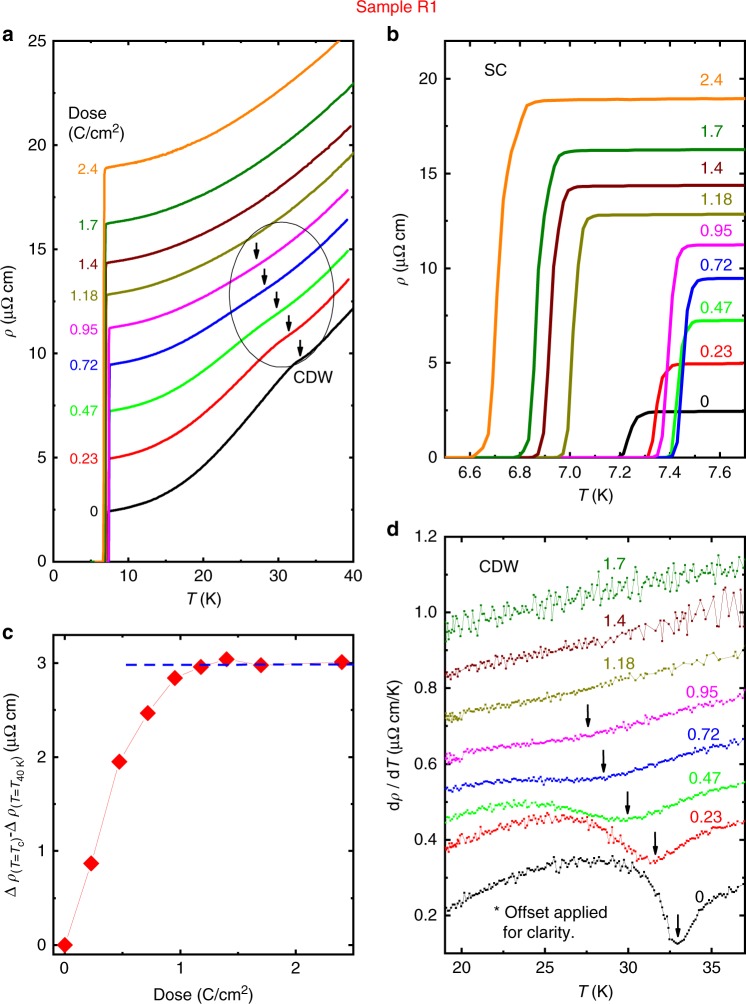
Resistivity measurement conducted on sample R1. **a** The temperature dependence of resistivity upon irradiation. Overall, the resistivity above the CDW transition was parallel-shifted upward suggesting that preservation of Matthiessen’s rule only occurs above the transition (see text for further discussion). **b** The zoom of superconducting transition area shows the increase and subsequent decrease of *T*_c_ upon irradiation. **c** Δ*ρ*_(*T*=7.6*K*)_ − Δ*ρ*_(*T*=40*K*)_ shows the disappearance of the CDW above 1.0 C cm^−2^. **d** The derivative of resistivity with respect to temperature manifests the location of the CDW transitions

Electron irradiation (maximum dose of 8.93 C cm^−2^) effectively introduces artificial disorder into the system, resulting in the substantial increase of residual resistivity in the normal state, as shown in Fig. 1. Above 40 K without long-range CDW order, the increase of resistivity is rather constant. However, near and below 40 K, a violation of Matthiesen’s rule was observed due to the presence of the CDW phase. For cases with high doses of irradiation (>1.0 C cm^−2^) where the CDW feature in resistivity was completely suppressed due to disorder, Matthiesen’s rule was obeyed over the entire temperature region of the normal state. To investigate how effectively the electron irradiation introduces disorder, the in-situ resistivity of sample R1 was measured during the irradiation at 22 K (inset of Fig. 1). It increases monotonically with increasing dose of irradiation. The blue arrows indicate when the irradiation stopped and room-temperature annealing occurred. About 30–40 % annealing occurred for each case. For each dose (blue arrows), the sample was removed from the irradiation chamber and moved to a different cryostat for measurement of the temperature-dependent resistivity as shown in Figs. 1 and 2.

**Fig. 3:**
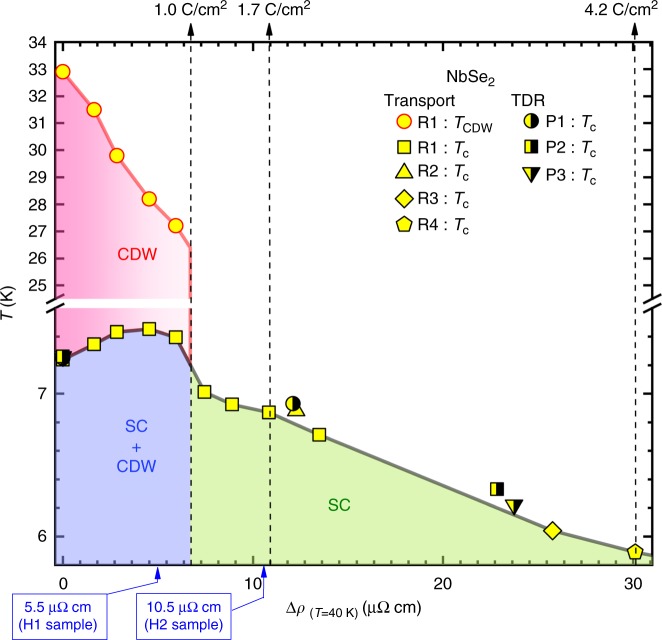
Temperature versus Δ*ρ*_(*T*=40*K*)_ phase diagram upon electron irradiation. For low doses of irradiation up to 1.0 C cm^−2^, *T*_c_ varies non-monotonically, whereas *T*_CDW_ monotonically decreases. When the resistivity feature of CDW disappears around 1.0 C cm^−2^ (shown in Fig. 2(b,d)), *T*_c_ suddenly drops by 0.3 K, indicating strong correlation between SC and CDW phases. Upon further irradiation, *T*_c_ monotonically decreases. The full phase diagram up to the highest dose of 8.93 C cm^−2^ is shown in Supplementary Figure 1. Hall resistivity is measured in two samples H1 and H2 (Fig. 6(a,b)). Two blue arrows in *x*-axis indicate their locations in the phase diagram, based on the increase in resistivity (Fig. 6(c,d))

With increasing irradiation dose, both the superconducting and CDW phases were substantially affected. As shown in Fig. 2(a), *T*_CDW_ (kink feature marked by an arrow) decreases with increasing irradiation and disappears after 1.0 C cm^−2^. The behaviour of the CDW feature is more clearly shown in a plot of *dρ*/*dT* vs. temperature (Fig. 2(d)). The important fact is that the feature associated with the CDW transition disappears at a finite temperature of 27 K, instead of continuing down to zero Kelvin. This result suggests the absence of a quantum critical point with disorder, in contrast to the previous pressure study by Feng et al.^29^. Figure 2(b) is an enlargement of the low-temperature part of Fig. 2(a) that shows the change of *T*_c_. It is clearly seen that *T*_*c*_ initially increases and then decreases upon irradiation. All the values of *T*_c_ and *T*_CDW_ for sample R1 are summarized in Fig. 3 along with *T*_c_’s from other samples (R2, R3, R4, P1, P2, P3). The *x*-axis of Fig. 3 is the increase of resistivity at 40 K, Δ*ρ*_(*T*=40*K*)_, upon irradiation (representing increased disorder). With increasing dose of irradiation up to 1.0 C cm^−2^ (Δ*ρ*_(*T*=40*K*)_ = 7.3 μΩ cm), *T*_c_ gradually increases from 7.25 K to 7.45 K, and then starts decreasing back to 7.3 K, whereas *T*_CDW_ monotonically decreases. Upon further irradiation, the feature associated with the CDW transition disappears and simultaneously *T*_c_ abruptly drops by 0.3 K, indicating strong correlation between SC and CDW phases. Note the mismatch between the maximum *T*_c_ and the disappearance of the CDW feature, suggesting a complex interplay between the two phases, potentially including other factors such as complicated Fermi surfaces. Upon further irradiation, *T*_c_ continues to decrease toward about 33% of its pristine value for the maximum electron dose of 8.93 C cm^−2^ (Δ*ρ*_(*T*=40*K*)_ = 61 μΩ cm) as shown in the full phase diagram in Supplementary Figure 1.

### Effect of electron irradiation on the London penetration depth

**Fig. 4:**
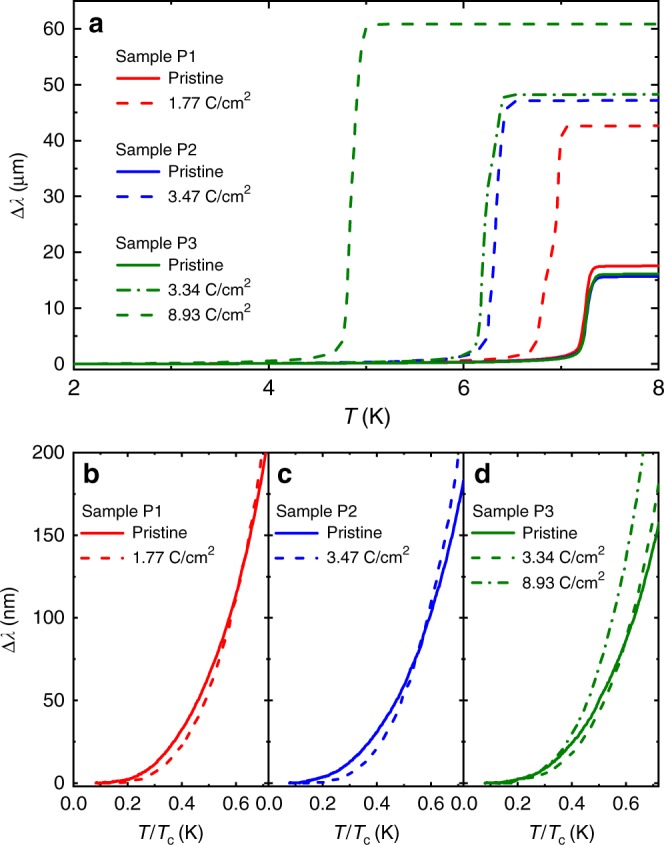
Temperature dependence of London penetration depth Δ*λ* upon electron irradiation. Results of three samples before and after irradiation: P1 (0 and 1.77 C cm^−2^), P2 (0 and 3.47 C cm^−2^), and P3 (0, 3.34, and 8.93 C cm^−2^). **a** Wide temperature span of Δ*λ* that shows a substantial decrease of *T*_c_ upon irradiation. **b**–**d** The low-temperature part of Δ*λ* of **b** P1, **c** P2, and **d** P3 samples before and after irradition. All data clearly show the saturating behaviour below 0.2 *T*/*T*_c_, supporting the presence of *s*-wave type superconducting gaps

Figure 4 shows the temperature dependence of the London penetration depth (Δ*λ*) of P1, P2, and P3 samples upon irradiation. The low-temperature saturation is clearly seen below 0.2 *T*/*T*_c_ for all samples before and after irradiation, suggesting the presence of *s*-wave type superconducting gaps. Interestingly, the saturation tendency gets stronger after irradiation, suggesting that the initial anisotropic gaps get more isotropic due to the gap-smearing effect of point-like disorder. Note that the irradiation doses shown correspond to residual resistivities Δ*ρ* beyond the initial enhancement of *T*_c_ due to competition with the CDW phase, so a uniform enhancement of the superconducting gap is not the main cause of the saturation in Δ*λ*. In addition, a substantial decrease of *T*_c_ from 7.25 K to 4.8 K (about 33% decrease) was found in sample P3 upon 8.93 C cm^−2^. These *T*_c_ suppressions of three samples (P1, P2, P3) are summarized in Fig. 3. Since we cannot directly obtain *ρ*_(*T*=40*K*)_ for P1, P2, and P3, we used the relation between dose and Δ*ρ* obtained from transport-measured samples (R1, R2, R3, and R4) as shown in Supplementary Figure 2(a). The substantial decrease of *T*_c_ and exponential-like saturation of Δ*λ* can be explained with multiband *s*-wave type superconducting gaps with some amount of interband coupling.

### Phase diagram upon electron irradiation

**Fig. 5:**
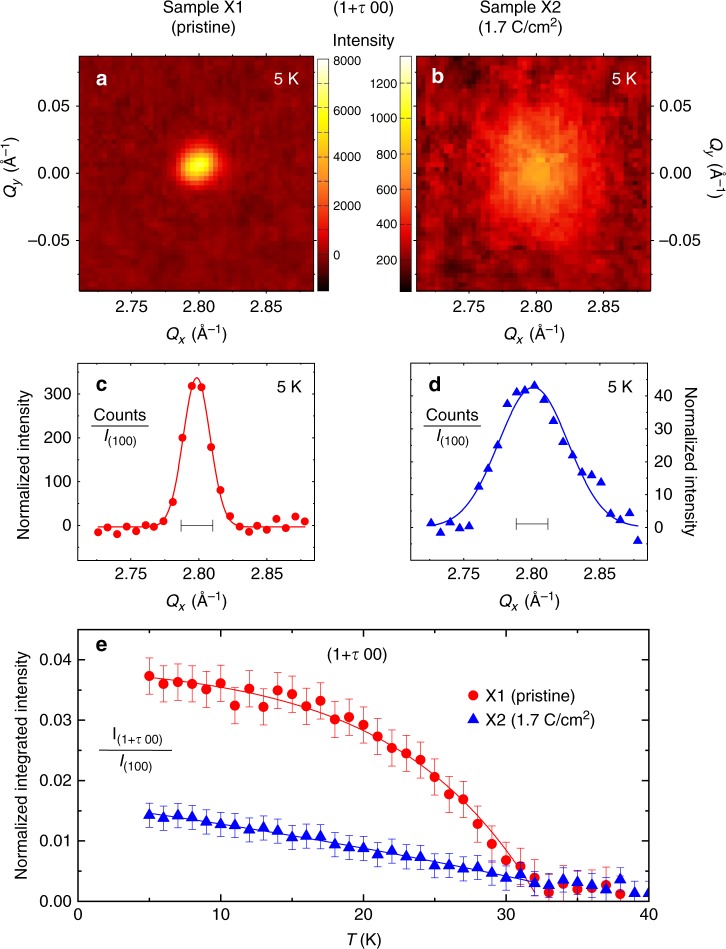
High-energy X-ray diffraction measurement of the CDW Bragg peak (1 + *τ* 0 0) with *τ* = 1/3. Results of two NbSe_2_ samples (X1: pristine, X2: 1.7 C cm^−2^). **a**,**b** Diffraction patterns of X1 and X2 recorded by the two-dimensional detector in the (*H K* 0) plane with intensity encoded in a linear color scale for each detector pixel. **c**,**d** Cuts along the longitudinal direction *Q*_*x*_, integrated along the transverse direction *Q*_*y*_ and normalized to the integrated intensity I_(100)_ of the weak (1 0 0) Bragg peak of the regular chemical lattice which is three orders of magnitude less intense than the strong (1 1 0) Bragg peak. The bars represent the instrumental resolution full-width half-maximum determined from the (1 0 0) Bragg peak. **e** Temperature dependence of the normalized integrated intensity of the CDW Bragg peak

**Fig. 6:**
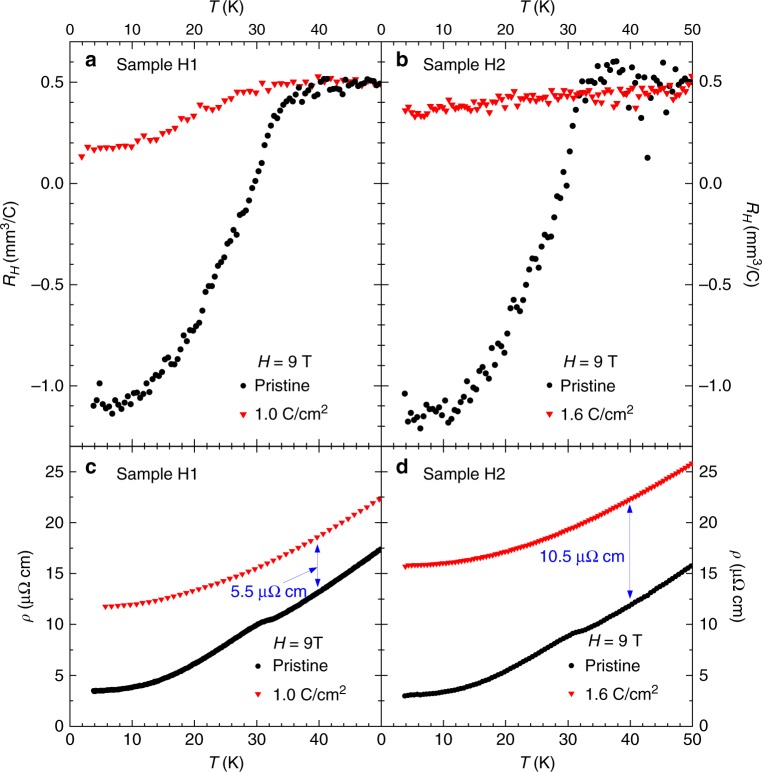
Temperature dependence of Hall resistivities of two NbSe_2_ samples measured under 9 T. **a** Sample H1 before and after 1.0 C cm^−2^ irradiation and **b** sample H2 before and after 1.6 C cm^−2^ irradiation. **c**,**d** In-field resistivities were also measured for both samples that clearly show the increase of disorder: Δ*ρ*_(*T*=40*K*)_ = 5.5 and 10.5 μΩ cm for samples H1 and H2, respectively

Figure 3 shows the temperature versus Δ*ρ*_(*T*=40*K*)_ phase diagram of SC and CDW upon electron irradiation obtained from seven samples. Upon initial irradiation up to 1.0 C cm^−2^, an anticorrelation of *T*_CDW_ and *T*_c_ was observed, which is most naturally interpreted in terms of strong competition between SC and CDW phases. However, after *T*_c_ reaches its maximum, both *T*_CDW_ and *T*_c_ decrease until the CDW phase abruptly disappears at a critical irradiation dose of 1.0 C cm^−2^, where *T*_c_ also drops discontinuously. The simplest explanation of the non-monotonic behaviour of *T*_c_ in the CDW coexistence phase is that the initial increase is due to the competition between SC and CDW phases. The effect of disorder on this competition was studied already by Grest et al.^33^ and Psaltakis et al.^42^. Within this weak coupling approach, non-magnetic disorder suppresses the CDW rapidly, and since the CDW order is competing with SC for the Fermi surface, *T*_c_ increases. Note that these theoretical calculations assume an isotropic *s*-wave gap; within their approximations, *T*_c_ would have saturated when the CDW order vanishs, due to Anderson’s theorem. However, it is clear from Fig. 3 that disorder continues to suppress *T*_*c*_ long after the CDW order is gone; this implies that the *s*-wave gaps have quite different amplitudes (and, possibly, anisotropy) and substantial interband pairing. Furthermore, as shown in Figs. 5 and 6, we found from the Hall resistivity and X-ray scattering that a short-range CDW phase survives long after the critical dose of 1.0 C cm^−2^.

### X-ray diffraction upon electron irradiation

Figure 5 shows the characterization of the CDW of samples X1 (pristine) and X2 (1.7 C cm^−2^) by high-energy X-ray diffraction. The structure of NbSe_2_ consists of layers of Nb atoms surrounded by six Se atoms and the Nb atoms located in the corners of the hexagonal unit cell^43^. The CDW displaces the six nearest Nb neighbors of every third Nb atom, yielding a superstructure with tripling of the unit-cell dimensions in both *a* and *b* directions, and corresponding propagation vectors (*τ* 0 0) and (0 *τ* 0) with *τ* = 1/3^44^. For both samples, the CDW Bragg peaks are observed in all measured Brillouin zones at low temperature. The CDW Bragg peaks are resolution limited, as illustrated in Fig. 5(a, c) for sample X1, whereas they show a significant broadening in panels (b) and (d) for sample X2, which is temperature-independent. Note that the intensity scales in panels (a) and (c) are about seven times larger than those in panels (b) and (d). From the peak widths determined by Gaussian fits to the cuts shown in Fig. 5(c, d), the correlation length of the CDW is estimated to be about 80 Å for the irradiated sample X2 and a lower limit of 200 Å for the pristine sample X1. The temperature dependence of the normalized integrated CDW Bragg peak intensity shown in Fig. 5(e) represents the square of the CDW order parameter and is clearly consistent with a second-order phase transition at *T*_CDW_ = 33 K and long-range order for sample X1. In contrast, the CDW intensity of the irradiated sample X2 increases continuously with decreasing temperature without a clear onset, indicating a crossover like behaviour. Together with the reduced correlation length, it is clear that the CDW manifests only short-range order in the irradiated sample X2, although the strength of the CDW is almost similar to the pristine sample X1 with the integrated Bragg peak intensity only reduced by 65% at low temperature.

The CDW appears in the same manner with comparable strength in irradiated samples but with a reduced correlation length. The irradiation induced defects likely form barriers or pinning centers for boundaries of the CDW state and prevent a coherently ordered state beyond these defects when the CDW state develops with decreasing temperature. The crossover-like temperature dependence of the short-range CDW without a clear onset observed for the sample X2 is consistent with the lack of a well-defined feature or signature of the CDW in transport measurements for samples with radiation levels above the critical dose of 1.0 C cm^−2^.

### Hall resistivities upon electron irradiation

Hall resistivities were measured for samples H1 and H2 as shown in Fig. 6(a, b). For sample H1, two measurements were conducted before and after irradiation with 1.0 C cm^−2^ and for sample H2 before and after irradiation with 1.6 C cm^−2^. First of all, the Hall resistivity of both pristine samples shows a sign change below *T*_CDW_ = 33 K, consistent with previous reports^45,46^. This indicates an increase of mobility in the CDW phase, consistent with previous reports in resistivity and Nernst effect^46^, due to an opening of the pseudogap^47^. The change of in-plane resistivity, *ρ*_*xx*_, as shown in panels (c) and (d), was used to accurately calibrate the amount of disorder, yielding Δ*ρ*_(*T*=40*K*)_ = 5.5 μΩ cm for sample H1 and 10.5 μΩ cm for H2. These values allow us to place the samples before and after the critical transition, respectively, as shown in the phase diagram (blue arrows in *x*-axis) of Fig. 3. The CDW transition is clearly seen for sample H1 in Fig. 6(a), consistent with the observation of a feature in the resistivity derivative. For sample H2, however, the feature at the CDW transition almost disappears in Fig. 6(b), although a slight slope change can be noticed at 30 K. This is consistent with the disappearance of the long-range CDW feature (resistivity) and the presence of a short-range CDW (X-ray scattering) above the critical dose. Another important fact is that the Hall resistivity above 40 K did not change upon irradiation. This implies that the defects introduced by electron irradiation do not change the electronic carrier density above 40 K, but only increase the scattering rate.

## Discussion

Evidence for an anisotropic superconducting gap in NbSe_2_ is provided, e.g., by STM measurements, which show a significantly broadened gap edge^12^. In addition, the flattening of the low-*T* penetration depth upon irradiation at doses corresponding to the pure superconducting phase is evident in Fig. 4. As the smallest gap in the system will determine the asymptotic low-*T* exponential dependence, it suggests that the disorder increases the minimum gap, i.e., gap averaging. The non-monotonic behaviour of *T*_c_ in the CDW/SC coexistence phase can therefore be understood simply by assuming that the effect of losing competition from the CDW is overcome by the gap averaging effect before the CDW disappears. It should be noted, however, that the behaviour with pressure is also non-monotonic^31^ In this case, the reason for the continued suppression of *T*_c_ is less obvious and the pressure dependence of the couplings of various phonons may be necessary to explain the complete behaviour quantitatively.

The possibility of a first-order transition at the disappearance of CDW order is also intriguing and recalls the question of the CDW mechanism. A simple Fermi surface nesting model^48^ fails to explain the CDW ordering vector and is therefore not appropriate for NbSe_2_^20,49^. Similarly, a saddle point-driven CDW instability proposed by Rice and Scott^50^ has been ruled out by ARPES^51^. However, a generalized Fermi surface nesting model, which includes the strong anisotropy in the electron-phonon matrix elements, does capture the correct CDW ordering vector^21,52^. The generalized Fermi-surface nesting model is still effectively a weak coupling model, where a strong momentum dependence of the electron-phonon matrix elements modifies the peak position of the charge susceptibility. Hence, from a weak coupling perspective, a disorder-driven first-order transition, as apparently observed here, appears to be a natural one. This is because the CDW is a Stoner-type instability, where with increasing disorder the charge susceptibility at the ordering vector should drop below a critical value corresponding to ordering.

Observation of a quantum critical point under pressure might be taken as evidence against the idea of a first-order transition^29^. However, one should keep in mind that pressure also changes the bare electronic structure, which does not happen in case of point-like impurities. Disorder is often thought to drive a first-order transition, e.g. in the manganites^53^. We note that Chatterjee et al.^22^ deduced a smooth decay of CDW order with chemical substitution, but in fact their data are entirely consistent with ours because of the relatively small number of doping levels studied in that work.

We cannot definitively rule out the possibility that the feature observed in transport, here identified as the signature of long-range CDW order, simply becomes too weak to observe because of broadening due to significant short-range fluctuations, as observed in Chatterjee et al.^22.^ However, our new observation of a concomitant abrupt drop in *T*_c_ suggests that a thermodynamic transition is indeed taking place at this critical value of disorder. Unlike incommensurate CDW systems, commensurate CDW transitions as in NbSe_2_ in the presence of quenched disorder may occur^54^. The ordered phase in such a situation breaks translational symmetry discretely, so that a second-order transition with exponents dependent on the order of the commensurability is allowed, but this can be preempted by a first-order transition, as apparently observed here.

We investigated the interplay between CDW and SC phases in 2*H*-NbSe_2_ by measuring in-plane resistivity, Hall resistivity, X-ray scattering, and London penetration depth before and after electron irradiation. Upon initial irradiation, *T*_c_ increased from 7.25 K to 7.45 K and then decreased, whereas *T*_CDW_ monotonically decreased. This indicates a complex interplay between two phases with potential other factors such as a complicated Fermi surface. Upon further irradiation, the feature associated with the CDW transition disappeared at finite temperature. When the CDW feature disappears, *T*_c_ abruptly drops by 0.3 K, indicating strong correlation between two phases and suggesting a first-order, disorder-driven phase transition. Further irradiation up to 8.93 C cm^−2^ effectively and monotonically decreased *T*_c_ down to 4.8 K (about 33% of its pristine value), suggestive of the averaging of an anisotropic *s*-wave superconducting order parameter. According to X-ray scattering and Hall resistivity studies, the short-range CDW is still present after the critical dose of ~1.0 C cm^−2^ (~7.3 μΩ cm), indicating that the effect of electron irradiation decreases the coherence of the CDW phase. The low-temperature penetration depth shows exponential-like behaviour below 0.2 *T*/*T*_c_ for all samples before and after irradiation. The combined results of resistivity and penetration depth can be explained with multiband anisotropic *s*-wave type superconducting gaps with some amount of interband coupling.

## Methods

### Crystal growth

The single crystals of 2*H*-NbSe_2_ from Bell Laboratories were grown using the iodine vapor transport technique and are known to be of high quality (RRR ~40). These are the samples from the same batch as used in Ref. ^23^.

### Four-probe resistivity

Four-probe measurements of in-plane resistivity were performed for four samples (R1, R2, R3, and R4). Samples for resistivity measurements had typical dimensions of (1–2) × 0.5 × (0.02–0.1) mm^3^. Electrical contacts to samples before irradiation were made by soldering 50 μm silver wires with indium and mechanically strengthened by silver paste as described elsewhere^55^. For in-situ resistivity measurements during the electron irradiation at 22 K, the R1 sample was mounted on a Kyocera chip and measured during irradiation.

### Hall resistivity

Simultaneous Hall effect and resistivity measurements were performed on samples H1 and H2 mounted in five-probe configuration using the same contact making technique as in resistivity measurements. Measurements were taken in a Quantum Design PPMS in constant magnetic fields + 9 T and − 9 T. The same samples with the same contacts were measured before and after irradiation, thus excluding geometric factor errors in quantitative comparison.

### London penetration depth

The in-plane London penetration depth Δ*λ* (*T*) of three other samples (P1, P2, and P3) was measured before and after irradiation using a self-oscillating tunnel-diode resonator technique^56–58^. The samples had typical dimensions of 0.5 × 0.5 × 0.03 mm^3^.

### X-ray diffraction

The high-energy X-ray diffraction study was performed at station 6-ID-D at the Advanced Photon Source, Argonne National Laboratory. The use of X-rays with an energy of 100.5 keV minimizes sample absorption and allows to probe the entire bulk of the sample using an incident beam with a size of 0.5 × 0.5 mm^2^, over-illuminating the sample. The samples were held on Kapton tape in a Helium closed-cycle refrigerator and Helium exchange gas was used. Extended regions of selected reciprocal lattice planes were recorded by a MAR345 image plate system positioned 1468 mm behind the sample as the sample was rocked through two independent angles up to ± 3.2° about axes perpendicular to the incident beam^59^.

### Electron irradiation

The 2.5 MeV electron irradiation was performed at the SIRIUS Pelletron facility of the Laboratoire des Solides Irradies at the Ecole Polytechnique in Palaiseau, France^60^. The acquired irradiation dose is conveniently measured in C cm^−2^, where 1 C cm^−2^ = 6.24 × 10^18^ electrons/cm^2^.

### Data availability

The authors declare that all data supporting the findings of this study are available within the article and its supplementary information files or from the corresponding author upon reasonable request.

## Electronic supplementary material

Supplementary Information

## References

[1] Dai P (2015). Antiferromagnetic order and spin dynamics in iron-based superconductors. Rev. Mod. Phys..

[2] Ghiringhelli G (2012). Long-range incommensurate charge fluctuations in (Y,Nd)Ba2Cu3O6+x. Science.

[3] Chang J (2012). Direct observation of competition between superconductivity and charge density wave order in YBa2Cu3O6.67. Nat. Phys..

[4] Achkar AJ (2012). Distinct charge orders in the planes and chains of ortho-iii-ordered YBa2Cu3O6+d superconductors identified by resonant elastic x-ray scattering. Phys. Rev. Lett..

[5] Comin R (2014). Charge order driven by fermi-arc instability in Bi2Sr2-xLaxCuO6 + d. Science.

[6] da Silva Neto EH (2014). Ubiquitous interplay between charge ordering and high-temperature superconductivity in cuprates. Science.

[7] Tabis W (2014). Charge order and its connection with Fermi-liquid charge transport in a pristine high-T_*c*_ cuprate. Nat. Commun..

[8] Gabovich AM, Voitenko AI, Annett JF, Ausloos M (2001). Charge- and spin-density-wave superconductors. Supercond. Sci. Technol..

[9] Wilson J, Salvo FD, Mahajan S (1975). Charge-density waves and superlattices in the metallic layered transition metal dichalcogenides. Adv. Phys..

[10] Moncton DE, Axe JD, DiSalvo FJ (1975). Study of superlattice formation in 2H-NbSe2 and 2H-TaSe2 by neutron scattering. Phys. Rev. Lett..

[11] Harper J, Geballe T, Salvo FD (1975). Heat capacity of 2H-NbSe2 at the charge density wave transition. Phys. Lett. A.

[12] Guillamon I, Suderow H, Guinea F, Vieira S (2008). Intrinsic atomic-scale modulations of the superconducting gap of 2H-NbSe2. Phys. Rev. B.

[13] Sooryakumar R, Klein MV (1980). Raman scattering by superconducting-gap excitations and their coupling to charge-density waves. Phys. Rev. Lett..

[14] Littlewood PB, Varma CM (1981). Gauge-invariant theory of the dynamical interaction of charge density waves and superconductivity. Phys. Rev. Lett..

[15] Littlewood PB, Varma CM (1982). Amplitude collective modes in superconductors and their coupling to charge-density waves. Phys. Rev. B.

[16] Méasson MA (2014). Amplitude higgs mode in the 2*h*−nbse_2_ superconductor. Phys. Rev. B.

[17] Grasset R (2018). Higgs-mode radiance and charge-density-wave order in 2H-NbSe2. Phys. Rev. B.

[18] Inglesfield JE (1980). Bonding and phase transitions in transition metal dichalcogenide layer compounds. J. Phys. C Solid State Phys..

[19] Varma CM, Simons AL (1983). Strong-coupling theory of charge-density-wave transitions. Phys. Rev. Lett..

[20] Weber F (2011). Extended phonon collapse and the origin of the charge-density wave in 2H-NbSe2. Phys. Rev. Lett..

[21] Flicker F, van Wezel J (2015). Charge order from orbital-dependent coupling evidenced by NbSe2. Nat. Commun..

[22] Chatterjee U (2015). Emergence of coherence in the charge-density wave state of 2H-NbSe_2_. Nat. Commun..

[23] Fletcher JD (2007). Penetration depth study of superconducting gap structure of 2H-NbSe2. Phys. Rev. Lett..

[24] Yokoya T (2001). Fermi surface sheet-dependent superconductivity in 2h-nbse2. Science.

[25] Boaknin E (2003). Heat conduction in the vortex state of nbse_2_: evidence for multiband superconductivity. Phys. Rev. Lett..

[26] Kiss T (2007). Charge-order-maximized momentum-dependent superconductivity. Nat. Phys..

[27] Borisenko SV (2009). Two energy gaps and fermi-surface “arcs” in NbSe2. Phys. Rev. Lett..

[28] Leroux M (2015). Strong anharmonicity induces quantum melting of charge density wave in 2H-NbSe2 under pressure. Phys. Rev. B.

[29] Feng Y (2012). Order parameter fluctuations at a buried quantum critical point. Proc. Natl Acad. Sci. USA.

[30] Berthier C, Molinié P, Jérome D (1976). Evidence for a connection between charge density waves and the pressure enhancement of superconductivity in 2H-NbSe2. Solid State Commun..

[31] Suderow H, Tissen VG, Brison JP, Martnez JL, Vieira S (2005). Pressure induced effects on the fermi surface of superconducting 2H-NbSe2. Phys. Rev. Lett..

[32] Mutka H (1983). Superconductivity in irradiated charge-density-wave compounds 2H-NbSe2, and 2H-TaSe2. Phys. Rev. B.

[33] Grest GS, Levin K, Nass MJ (1982). Impurity and fluctuation effects in charge-density-wave superconductors. Phys. Rev. B.

[34] Hirschfeld PJ, Goldenfeld N (1993). Effect of strong scattering on the low-temperature penetration depth of a *d*-wave superconductor. Phys. Rev. B.

[35] Wang Y, Kreisel A, Hirschfeld PJ, Mishra V (2013). Using controlled disorder to distinguish *s*_±_ and *s*_++_ gap structure in fe-based superconductors. Phys. Rev. B.

[36] Cho K (2016). Energy gap evolution across the superconductivity dome in single crystals of (Ba1-xKx)Fe2As2. Sci. Adv..

[37] Prozorov R (2014). Effect of electron irradiation on superconductivity in single crystals of Ba(Fe_1−*x*_Ru_*x*_)_2_As_2_ (x = 0.24). Phys. Rev. X.

[38] Cho K, Kon′czykowski M, Teknowijoyo S, Tanatar MA, Prozorov R (2018). Using electron irradiation to probe iron-based superconductors. Supercond. Sci. Technol..

[39] Mizukami Y (2014). Disorder-induced topological change of the superconducting gap structure in iron pnictides. Nat. Commun..

[40] Strehlow CP (2014). Comparative study of the effects of electron irradiation and natural disorder in single crystals of SrFe2(AsP)2 superconductor (*x* = 0.35). Phys. Rev. B.

[41] Psaltakis GC (1984). Non-magnetic impurity effects in charge-density-wave superconductors. J. Phys. C Solid State Phys..

[42] Marezio M, Dernier P, Menth A, Hull G (1972). The crystal structure of NbSe2 at 15 k. J. Solid State Chem..

[43] Malliakas CD, Kanatzidis MG (2013). Nb-nb interactions define the charge density wave structure of 2H-NbSe2. J. Am. Chem. Soc..

[44] Yamaya K, Sambongi T (1972). Low-temperature crystal modification and the superconductive transition temperature of nbse2. Solid State Commun..

[45] Bel R, Behnia K, Berger H (2003). Ambipolar nernst effect in NbSe2. Phys. Rev. Lett..

[46] Evtushinsky DV (2008). Pseudogap-driven sign reversal of the hall effect. Phys. Rev. Lett..

[47] Wilson JA (1977). Charge-density waves in the 2H-NbSe2 family: Action on the fermi surface. Phys. Rev. B.

[48] Valla T (2004). Quasiparticle spectra, charge-density waves, superconductivity, and electron-phonon coupling in 2H-NbSe2. Phys. Rev. Lett..

[49] Rice TM, Scott GK (1975). New mechanism for a charge-density-wave instability. Phys. Rev. Lett..

[50] Rossnagel K (2001). Fermi surface of 2H-NbSe2 and its implications on the charge-density-wave mechanism. Phys. Rev. B.

[51] Doran NJ (1978). A calculation of the electronic response function in 2H-NbSe2 including electron-phonon matrix element effects. J. Phys. C Solid State Phys..

[52] Dagotto E (2005). Complexity in strongly correlated electronic systems. Science.

[53] Nie L, Tarjus G, Kivelson SA (2014). Quenched disorder and vestigial nematicity in the pseudogap regime of the cuprates. Proc. Natl. Acad. Sci..

[54] Tanatar MA (2016). Origin of the resistivity anisotropy in the nematic phase of fese. Phys. Rev. Lett..

[55] Prozorov R, Giannetta RW, Carrington A, Araujo-Moreira FM (2000). Meissner-london state in superconductors of rectangular cross section in a perpendicular magnetic field. Phys. Rev. B.

[56] Prozorov R, Giannetta RW (2006). Magnetic penetration depth in unconventional superconductors. Supercond. Sci. Technol..

[57] Prozorov R, Kogan VG (2011). London penetration depth in iron-based superconductors. Rep. Progress. Phys..

[58] Kreyssig A (2007). Crystallographic phase transition within the magnetically ordered state of Ce2Fe17. Phys. Rev. B.

[59] van der Beek CJ (2013). Electron irradiation of co, ni, and p-doped BaFe2As2—type iron-based superconductors. J. Phys. Conf. Ser..

[60] Efremov DV, Korshunov MM, Dolgov OV, Golubov AA, Hirschfeld PJ (2011). Disorder-induced transition between *s*_±_ and *s*_++_ states in two-band superconductors. Phys. Rev. B.

