# Supplementary material for: Using controlled disorder to probe the interplay between charge order and superconductivity in NbSe2
Source: Nat Commun. 2018 Jul 18;9:2796. doi: 10.1038/s41467-018-05153-0 (PMC6052160; doi:10.1038/s41467-018-05153-0)
Supplement: Supplementary file 1 — Supplementary Information [file 41467_2018_5153_MOESM1_ESM.pdf]

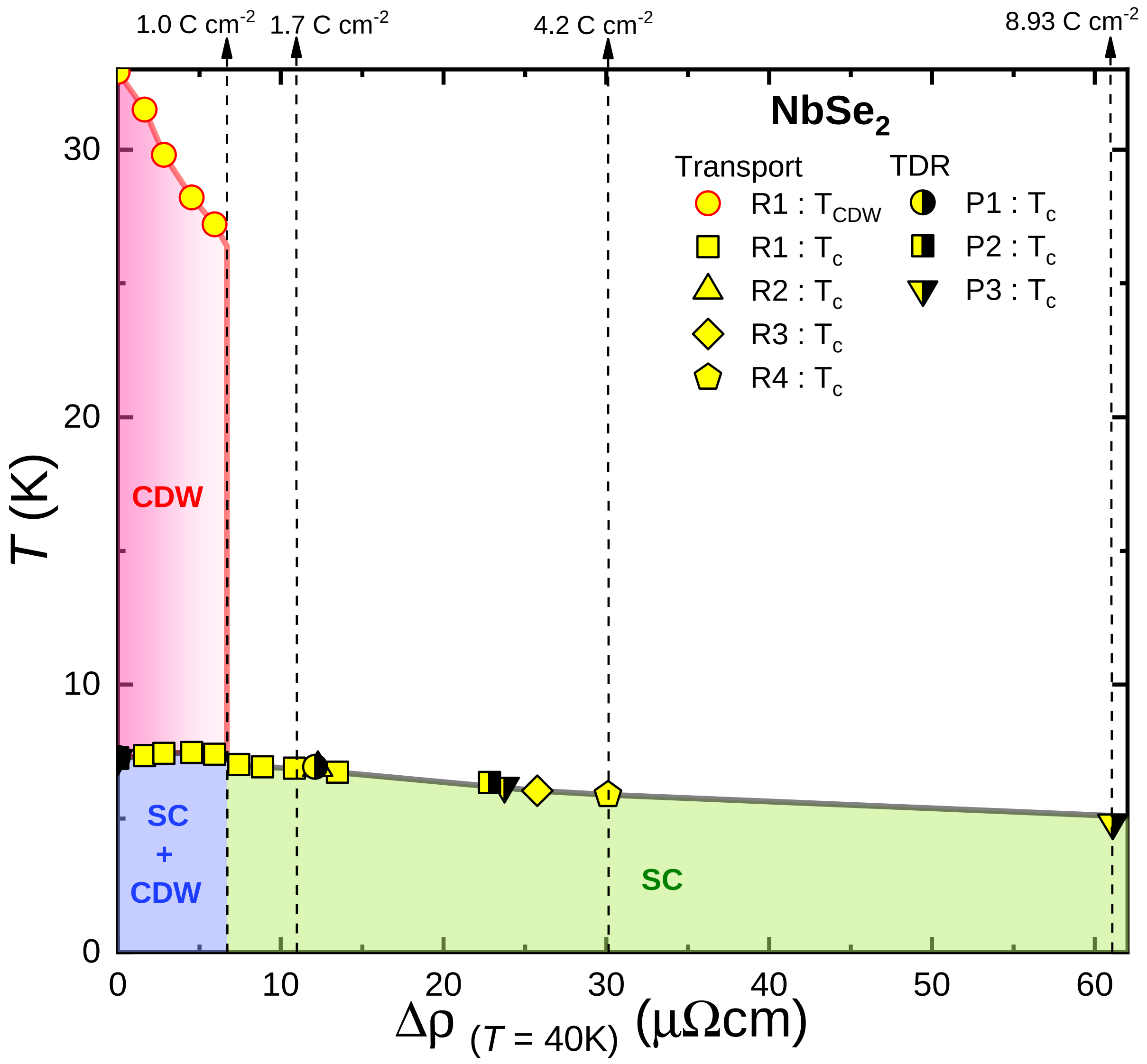

Supplementary Figure 1. **Full phase diagram of Fig. 4**. The highest dose is 8.93 C cm^{-2} ($\Delta\rho_{(T=40K)} = 61$ $\mu\Omega$cm).

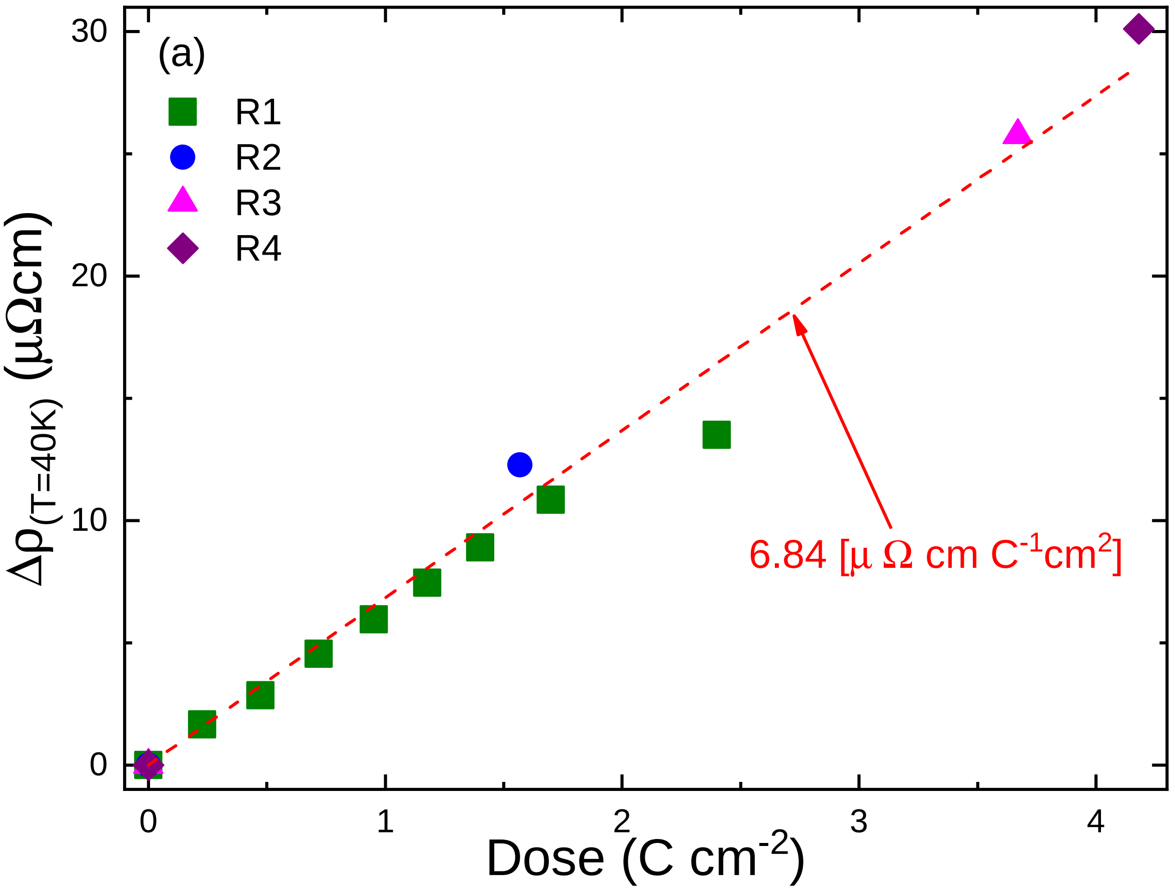

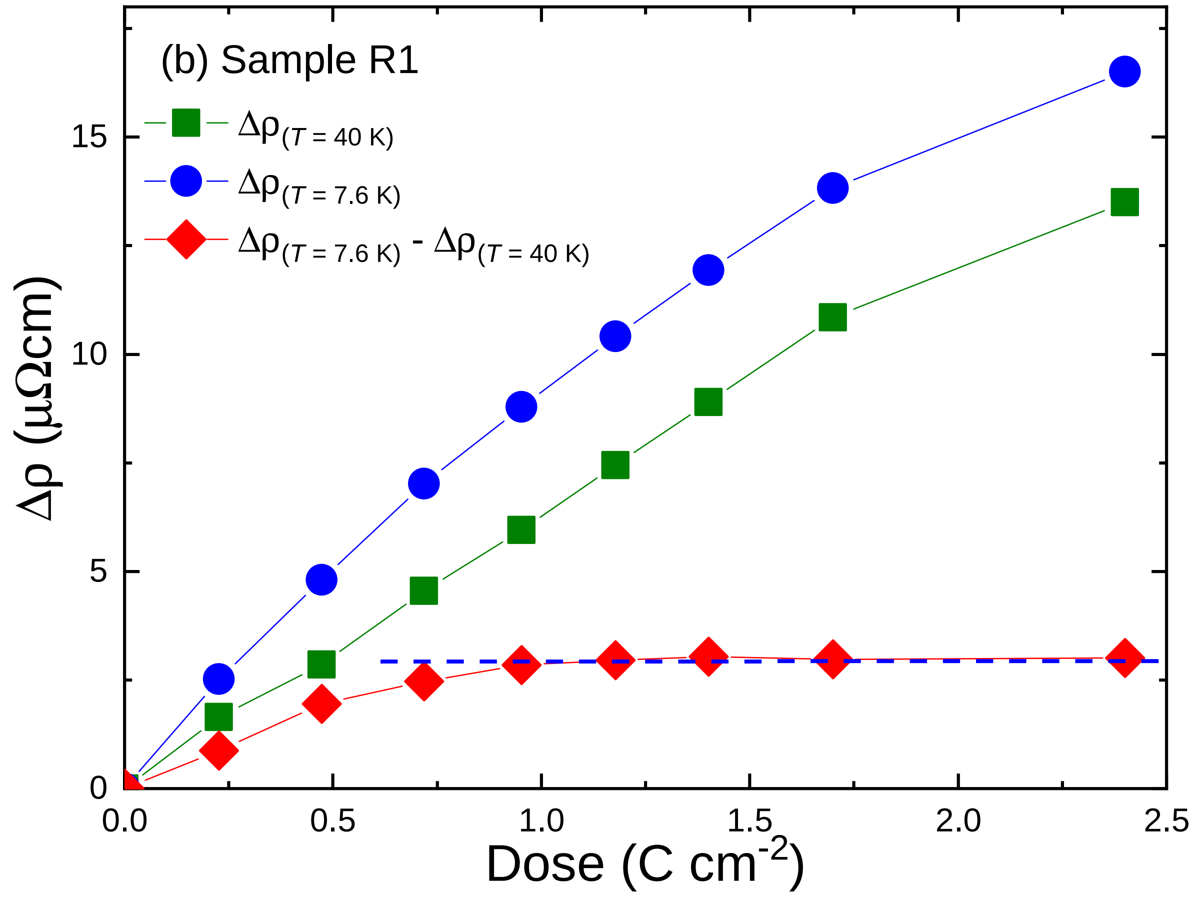

Supplementary Figure 2. **Relation between dose (C cm^{-2}) and $\Delta\rho_{(T=40K)}$ for sample R1.** (a) Dose (C cm^{-2}) versus $\Delta\rho_{(T=40K)}$. (b) Comparison between $\Delta\rho_{(T=40K)}$ and $\Delta\rho_{(T=7.6K)}$ that shows the suppression of the CDW above 1.0 C cm^{-2}. Refer to Fig. 2 (c).
